# The Pharmacological Chaperone AT2220 Increases Recombinant Human Acid α-Glucosidase Uptake and Glycogen Reduction in a Mouse Model of Pompe Disease

**DOI:** 10.1371/journal.pone.0040776

**Published:** 2012-07-18

**Authors:** Richie Khanna, John J. Flanagan, Jessie Feng, Rebecca Soska, Michelle Frascella, Lee J. Pellegrino, Yi Lun, Darlene Guillen, David J. Lockhart, Kenneth J. Valenzano

**Affiliations:** Amicus Therapeutics Inc, Cranbury, New Jersey, United States of America; University of Florida, United States of America

## Abstract

Pompe disease is an inherited lysosomal storage disease that results from a deficiency in the enzyme acid α-glucosidase (GAA), and is characterized by progressive accumulation of lysosomal glycogen primarily in heart and skeletal muscles. Recombinant human GAA (rhGAA) is the only approved enzyme replacement therapy (ERT) available for the treatment of Pompe disease. Although rhGAA has been shown to slow disease progression and improve some of the pathophysiogical manifestations, the infused enzyme tends to be unstable at neutral pH and body temperature, shows low uptake into some key target tissues, and may elicit immune responses that adversely affect tolerability and efficacy. We hypothesized that co-administration of the orally-available, small molecule pharmacological chaperone AT2220 (1-deoxynojirimycin hydrochloride, duvoglustat hydrochloride) may improve the pharmacological properties of rhGAA via binding and stabilization. AT2220 co-incubation prevented rhGAA denaturation and loss of activity *in vitro* at neutral pH and 37°C in both buffer and blood. In addition, oral pre-administration of AT2220 to rats led to a greater than two-fold increase in the circulating half-life of intravenous rhGAA. Importantly, co-administration of AT2220 and rhGAA to *GAA* knock-out (KO) mice resulted in significantly greater rhGAA levels in plasma, and greater uptake and glycogen reduction in heart and skeletal muscles, compared to administration of rhGAA alone. Collectively, these preclinical data highlight the potentially beneficial effects of AT2220 on rhGAA *in vitro* and *in vivo*. As such, a Phase 2 clinical study has been initiated to investigate the effects of co-administered AT2220 on rhGAA in Pompe patients.

## Introduction

Pompe disease (OMIM 23200), also referred to as glycogen storage disease type II or acid maltase deficiency, is a lysosomal storage disease (LSD) caused by mutations in the gene (*GAA*) that encodes the lysosomal hydrolase acid α-glucosidase (GAA) [1]–[2]. Deficiency of GAA activity results in progressive accumulation and deposition of glycogen in the lysosomes of heart, skeletal muscles, and other tissues. The disease encompasses a broad spectrum of phenotypes that range from the severe infantile-onset form to more slowly progressing late-onset forms [2]–[5]. Early or infantile-onset Pompe disease occurs before 12 months of age, has a rapid rate of progression, and is typically characterized by severe muscle weakness, frequent respiratory infections, hepatomegaly, massive cardiomegaly, cardiomyopathy, and cardiorespiratory failure that usually results in death between 1 and 2 years of age [1], [3]. Late-onset forms of the disease occur between childhood and adulthood, have a slower rate of progression, and are typically characterized by musculoskeletal and pulmonary involvement that lead to progressive weakness and respiratory insufficiency [1], [3]–[5].

Enzyme replacement therapy (ERT) is currently the primary treatment for Pompe disease [6]. ERT is based on the intravenous administration of recombinant human GAA (rhGAA), of which Myozyme® and Lumizyme® (alglusidase alfa; Genzyme Corporation, Cambridge, MA) are the only two approved products. Infantile Pompe patients treated with ERT show improvements on cardiac hypertrophy and motor skills, with a substantial increase in life-span [5], [7]–[9]. Late-onset patients have shown mild improvements in motor and respiratory functions following therapy with ERT, though clinical efficacy in these patients still requires long-term evaluation [10]–[11]. Despite the clinical benefits of ERT, a number of reports suggest that correction of the skeletal muscle phenotype is particularly challenging, and that not all patients respond equally well to treatment [2], [7], [12]–[13]. These limitations are at least partially due to insufficient targeting/uptake into disease-relevant tissues, as well as poor tolerability due to severe ERT-mediated anaphylactic and immunologic reactions [5], [11], [14]–[18].

Small molecule pharmacological chaperones (PCs) have been proposed as a potential therapy for Pompe disease [19]–[22]. The iminosugar 1-deoxynojirimycin hydrochloride (AT2220, duvoglustat hydrochloride) acts as a PC for GAA, selectively and reversibly binding and stabilizing the endogenous enzyme, facilitating proper protein folding and trafficking to lysosomes [21]. AT2220 has been shown to increase endogenous levels of many different mutant forms of GAA [21]–[22]. PCs have also been identified that selectively bind, stabilize, and increase the levels of the mutated enzymes that are associated with several other LSDs, including Gaucher, Tay-Sachs, Sandhoff, GM1-gangliosidosis, and Fabry disease [23].

Recently, the ability of PCs to improve the physical stability, and to increase cellular and tissue uptake, has been demonstrated for several exogenous recombinant enzymes that are used to treat LSDs. Specifically, the PCs AT1001 (deoxygalactonojirimycin), isofagomine, and *N*-butyl-deoxynojirimycin were shown to increase the *in vitro* cellular uptake and/or *in vivo* tissue uptake of the recombinant enzymes used to treat Fabry [24]–[26], Gaucher [27], and Pompe [24] diseases, respectively. Furthermore, AT1001 co-administration with recombinant human α-galactosidase A leads to greater substrate reduction in cells and tissues of Fabry mice compared to administration of enzyme alone [25]–[26].

Here, we demonstrate that AT2220 stabilizes rhGAA *in vitro*, minimizing protein denaturation and inactivation at neutral pH and physiological temperature. Studies in rats and *GAA* knock-out mice indicate that oral pre-administration of AT2220 increases the circulating half-life of rhGAA, and leads to significant increases in rhGAA activity in disease-relevant tissues. Most importantly, we show that AT2220-mediated increases in rhGAA tissue levels translate to greater glycogen reduction compared to administration of rhGAA alone, thus indicating a “boost” in the net lysosomal activity from the exogenous recombinant enzyme. Taken together, these data indicate that AT2220 can increase the stability and improve the pharmacokinetic properties of rhGAA, thereby leading to increased tissue enzyme activity and greater substrate reduction. As such, a Phase 2 clinical study has been initiated to investigate the effects of co-administered AT2220 on rhGAA in Pompe patients.

## Materials and Methods

### Materials

All cell culture reagents were purchased from Invitrogen (Carlsbad, CA), except for characterized fetal bovine serum (FBS), which was purchased from HyClone (Waltham, MA). AT2220 (1-deoxynojirimycin hydrochloride, duvoglustat hydrochloride) was synthesized by WuXi PharmaTech (Shanghai, China). Recombinant human acid α-glucosidse (rhGAA; alglucosidase alfa; Myozyme®) was purchased from Genzyme Corp. (Cambridge, MA). The rabbit anti-human GAA polyclonal antibody, FL059, was a kind gift of Dr. Barry Byrne (University of Florida, Gainesville). Horseradish peroxidase-conjugated goat anti-rabbit IgG secondary antibody was purchased from ThermoPierce (Jackson Immunosearch Labs, West Grove, PA). *GAA* knock-out (KO) mice were kindly provided by Dr. Barry Byrne. Wild-type C57BL/6 mice and Sprague-Dawley rats (carotid artery cannulated) were purchased from Taconic Farms (Germantown, NY). Animal husbandry and all experiments were conducted under Institutional Animal Care and Use Committee approved protocols. All other reagents were purchased from Sigma Aldrich (St. Louis, MO) unless noted otherwise.

### Thermal Stability

**Figure 1:**
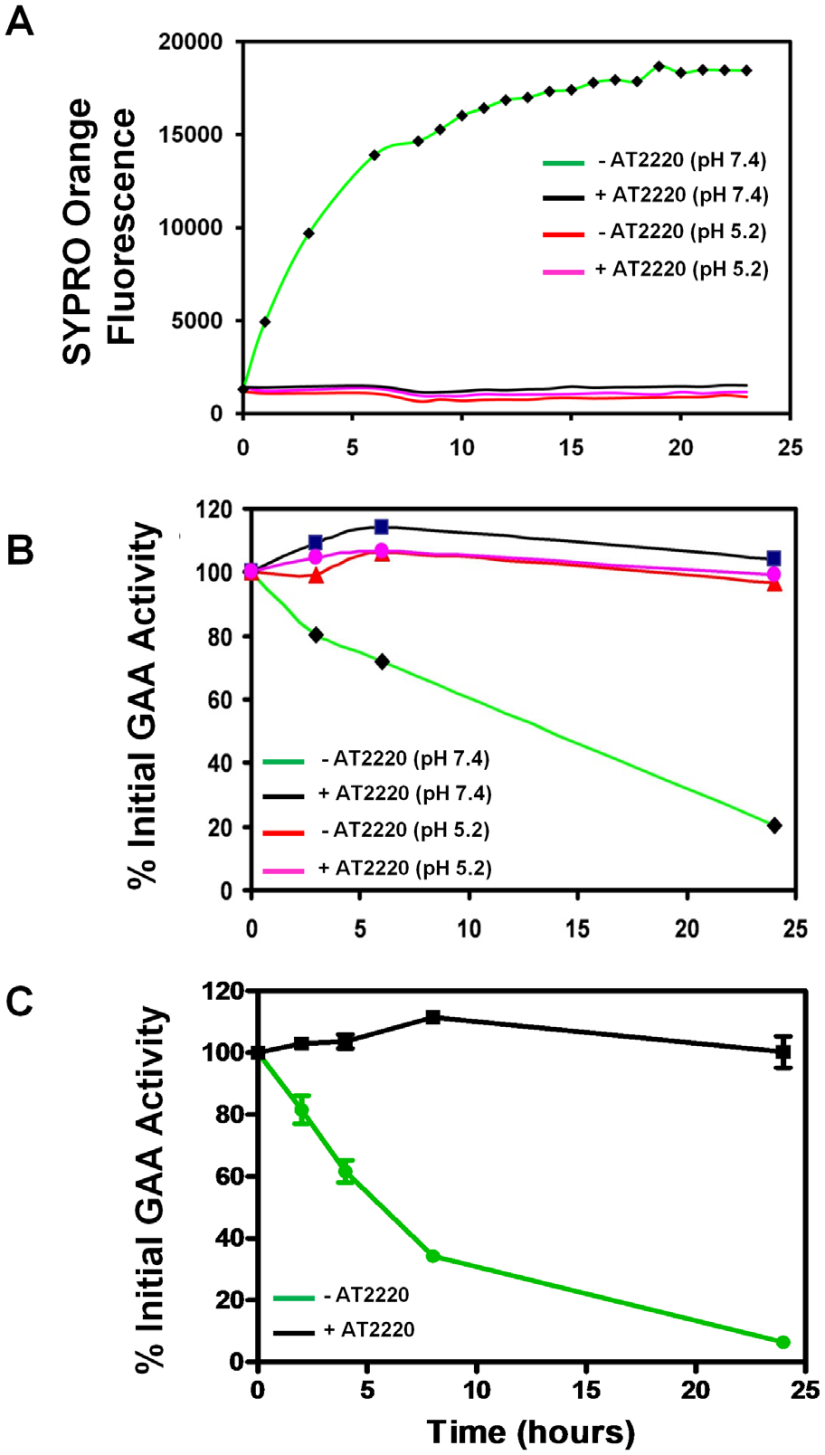
AT2220 increases the physical stability of rhGAA in vitro. (**A**) Time course of rhGAA denaturation in neutral and acidic buffer at 37°C in the absence and presence of 50 µM AT2220. Denaturation was monitored by changes in the fluorescence of SYPRO Orange as a function of time. (**B**) Time course of rhGAA inactivation (*i.e.* loss of activity) in neutral and acidic buffer at 37°C in the absence and presence of 50 µM AT2220. (**C**) Time course of rhGAA inactivation (*i.e.* loss of activity) in human whole blood at 37°C in the absence and presence of 50 µM AT2220. In both (**B**) and (**C**), GAA enzyme activity was determined at the indicated time points using the fluorogenic substrate 4-MUG. To obtain relative enzyme activity levels, measurements at the various time points were compared to the activity at the zero time point.

The stability of rhGAA was assessed using a modified fluorescence thermostability assay [28] on a Realplex Mastercycler qRT-PCR system (Eppendorf, Hamburg, Germany) in either neutral pH buffer (25 mM sodium phosphate, 150 mM sodium chloride, pH 7.4) or acidic pH buffer (25 mM sodium acetate, 150 mM sodium chloride, pH 5.2). For **Fig. 1A**, rhGAA (2.5 µg) was combined with SYPRO Orange and 50 µM AT2220 in a final reaction volume of 25 µL. For time-dependent denaturation assay, reactions were incubated at 37°C for up to 24 hours, with SYPRO Orange fluorescence intensity monitored at the indicated time points.

### Enzyme Inactivation in Buffer or Whole Blood

For time-dependent loss of activity assays shown in **Figs. 1B **and **1C**, rhGAA (500 nM) was incubated with or without 50 µM AT2220 for 15 minutes on ice in various pH buffers (neutral or acidic) or whole blood (Lampire; Pipersville, PA). Reactions were then transferred to 37°C, with aliquots removed at the indicated time points and measured for GAA activity. For GAA activity measurements, samples were diluted 100-fold in 50 mM potassium acetate (pH 4.0) prior to a further 10-fold dilution into a 96-well plate containing reaction buffer (50 mM potassium acetate, 3.3 mM 4-methylumbelliferryl-α-D-glucopyranoside (4-MUG), pH 4.0). Activity assays were performed for one hour at 37°C and stopped by the addition of an equal volume of 0.5 M sodium carbonate (pH 10.5). Liberated 4-MU was measured on a Victor^3^ plate reader (Perkin Elmer, Waltham, MA) at 355 nm excitation and 460 nm emission. Normalized fluorescence data were plotted as a function of time.

### AT2220/rhGAA Co-administration Studies in Rats

**Figure 2:**
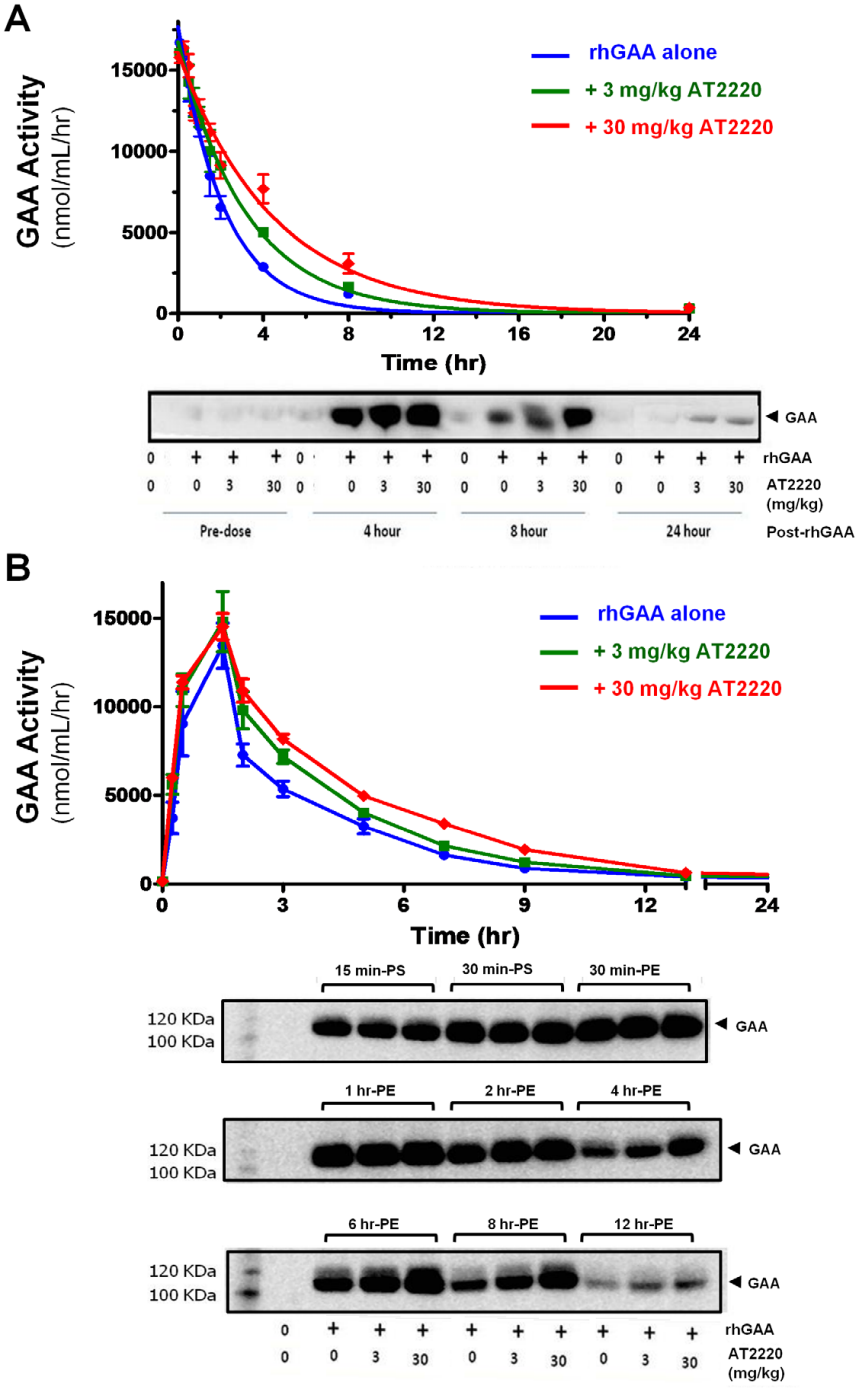
AT2220 increases the circulating half-life of rhGAA in rats. (**A**) Eight-week old male Sprague Dawley rats were administered vehicle (water) or AT2220 (3 or 30 mg/kg) via oral gavage. Thirty minutes later, vehicle (saline) or rhGAA (10 mg/kg) was administered via bolus tail vein injection. Blood was collected at the indicated time points, and GAA activity (upper panel) and protein levels (lower panel) were measured in plasma as described in ‘Materials and Methods’. (**B**) Eight-week old male Sprague Dawley rats were administered vehicle (water) or AT2220 (3 or 30 mg/kg) via oral gavage. Thirty minutes later, vehicle (saline) or rhGAA (10 mg/kg) was administered via 60-minute intravenous infusion. Blood was collected at the indicated time points, and GAA activity (upper panel) and protein levels (lower panel) were measured in plasma. *PS*: post-start infusion; *PE*: post-end infusion. In both (**A**) and (**B**), each time point represents the mean±SEM of the activity measured from 3 rats; each lane on the Western blot contains plasma from a single rat, and is representative of two rats in each group.

The rat co-administration studies were designed to investigate the effects of 3 and 30 mg/kg AT2220 on the circulating half-life of 10 mg/kg rhGAA when administered as a bolus tail vein injection or as an intravenous infusion. This dose of rhGAA provided significant activity above the endogenous GAA levels in normal rat plasma, and minimized the quantity of rhGAA required to complete these studies. Furthermore, 3 and 30 mg/kg AT2220 yield plasma exposures in rats that are comparable to those seen in humans following oral administration of 50 and 600 mg AT2220, respectively [29]. For IV bolus study (**Fig. 2A**), eight-week old male Sprague-Dawley rats were administered either vehicle (water) or AT2220 (3 or 30 mg/kg) via oral gavage. Thirty minutes later, vehicle (saline) or rhGAA (10 mg/kg) was administered via bolus tail vein injection. Whole blood was collected into lithium heparin tubes from the carotid artery cannula at the indicated time points. Plasma was collected by centrifuging blood at 2700 g for 10 minutes at 4°C, and was used for measurement of GAA activity as described below. For the infusion study (**Fig. 2B**), the same procedure was followed, except that rhGAA was administered as an intravenous infusion over 60 minutes at a rate of 5 mL/kg/hour. Comparative descriptions of the various doses, routes, and regimens for rhGAA and AT2220 administration are presented in **Table 1**.

**Table 1:** Description of the different doses, routes, and regimens utilized for rhGAA and AT2220 administration.

Figure	Species	Age (weeks)	rhGAA (mg/kg)	AT2220(mg/kg)	Route of Administration/Timing	Number of Administrations
2A	SD rats	8	10	3 and 30	AT2220 PO 30 minutes prior to rhGAA IV bolus	Single
2B	SD rats	8	10	3 and 30	AT2220 PO 30 minutes prior to rhGAA IV infusion	Single
3, 4, and 6	*GAA* KO mice	12	20	30	AT2220 PO 30 minutes prior to rhGAA IV bolus	Four bi-weekly
5	*GAA* KO mice	12	20	10 and 30	AT2220 PO 30 minutes prior to rhGAA IV bolus	Four bi-weekly

SD, Sprague Dawley.

### AT2220/rhGAA Co-administration Studies in GAA KO Mice

**Figure 3:**
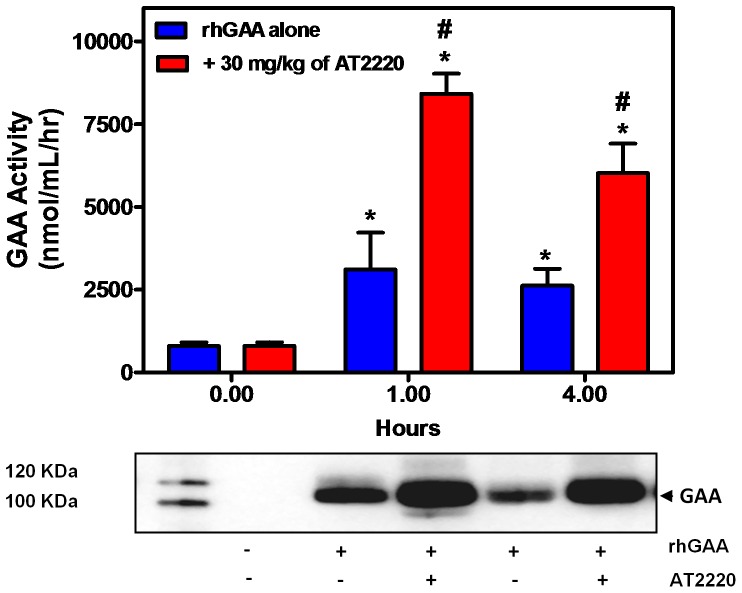
AT2220 increases the circulating levels of rhGAA in GAA KO mice. Twelve-week old male *GAA* KO mice were administered vehicle (water) or AT2220 (30 mg/kg) via oral gavage once every other week for 8 weeks. Thirty minutes after each AT2220 oral administration, vehicle (saline) or rhGAA (20 mg/kg) was administered via bolus tail vein injection. Blood was collected after the last (*i.e.,* 4^th^) rhGAA administration and, GAA activity (upper panel) and protein levels (lower panel) were measured in plasma as described in ‘Materials and Methods’. Each bar represents the mean±SEM of the GAA activity measured from 5 mice per group. Statistically significant increases were seen in plasma GAA activity compared to baseline (*p<0.05, t-test) and compared to rhGAA administration alone (#p<0.05, t-test). Each lane on the Western blot contains plasma from a single mouse, and is representative of two mice in each group.

Studies in *GAA* KO mice were designed to investigate the effects of a higher, clinically-relevant dose of rhGAA (20 mg/kg), as the focus was primarily efficacy (measured by rhGAA tissue uptake and glycogen reduction). The doses of AT2220 were limited to 10 and 30 mg/kg, which yield plasma exposures in mice that are comparable to those seen in humans following oral administration of 200 and 600 mg AT2220, respectively [29]. Our unpublished data suggest that even larger doses of AT2220 result in high and sustained muscle concentrations that have the potential for long-term inhibition of rhGAA; hence, doses above 30 mg/kg were not investigated [29]. In the studies shown in **Figs. 3** **,** **4** **,** **5**, and **6**, 12-week old male *GAA* KO mice were administered vehicle (water) or AT2220 (10 and/or 30 mg/kg) via oral gavage once every other week for 8 weeks. Thirty minutes after AT2220, rhGAA (20 mg/kg) was administered via bolus tail vein injection. Diphenhydramine (DPH; 10 mg/kg) was administered intraperitoneally 10 minutes before the 3^rd^ and 4^th^ rhGAA injections to minimize hypersensitivity reactions to rhGAA. Tissues were collected 7 or 21 days following the last rhGAA administration. In all studies, heart, diaphragm, tongue, skin (shaved and removed from the lower ventral side of the neck), hindlimbs (for isolation of quadriceps and gastrocnemius), and forelimbs (for isolation of triceps) were quickly removed, rinsed in cold phosphate-buffered saline, blotted dry, and stored on dry ice. Samples of heart and quadriceps were stored in fixatives as described below for immunohistochemical analysis. Comparative descriptions of the various doses, routes, and regimens for rhGAA and AT2220 administration are presented in **Table 1**.

**Figure 4:**
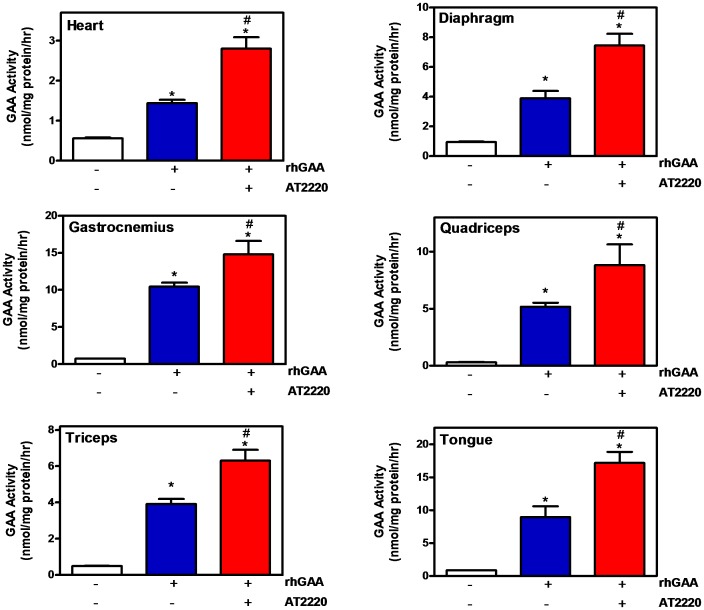
Co-administration of AT2220 promotes greater tissue uptake of rhGAA in GAA KO mice. Twelve-week old male *GAA* KO mice were administered vehicle (water) or AT2220 (30 mg/kg) via oral gavage once every other week for 8 weeks. Thirty minutes after each AT2220 oral administration, vehicle (saline) or rhGAA (20 mg/kg) was administered via bolus tail vein injection. Mice were euthanized 7 days after the last (*i.e.*, 4^th^) rhGAA administration and tissue GAA activity was measured as described in ‘Materials and Methods’. Each bar represents the mean±SEM of the GAA activity measured from 5 mice per group. Statistically significant increases were seen in GAA activity compared to baseline (*p<0.05, t-test) and compared to rhGAA administration alone (#p<0.05, t-test). For comparison, GAA levels in wild-type C57BL/6 mice were 15±2, 16±0.6, 21±3, 18±2, 11±2, and 25±3 nmol/mg protein/hr in heart, diaphragm, gastrocnemius, quadriceps, triceps, and tongue, respectively (mean±SEM of 7 mice).

**Figure 5:**
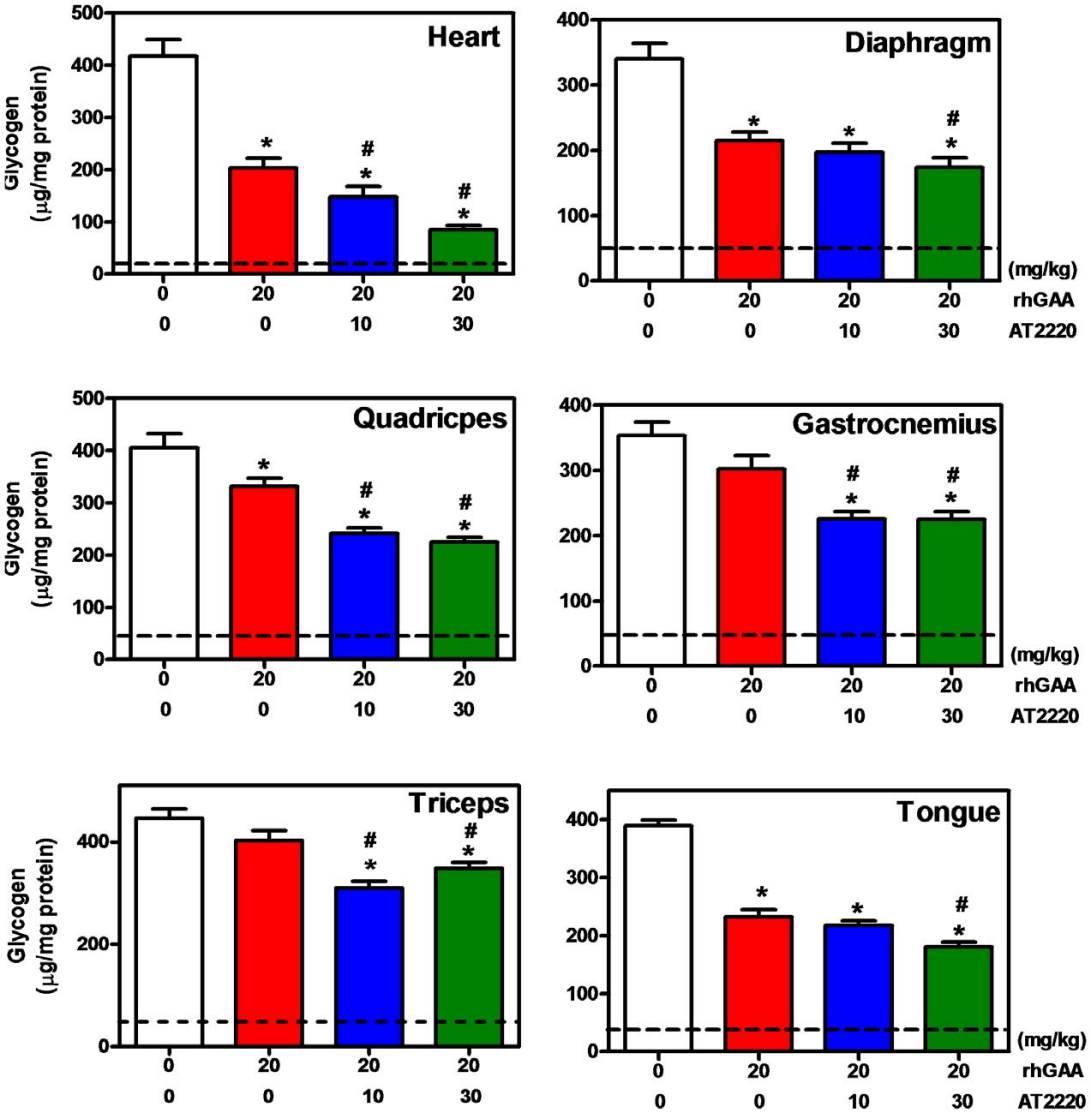
Co-administration of AT2220 promotes greater tissue glycogen reduction in GAA KO mice. Twelve-week old male *GAA* KO mice were administered vehicle (water) or AT2220 (10 or 30 mg/kg) via oral gavage once every other week for 8 weeks. Thirty minutes after each AT2220 administration, vehicle (saline) or rhGAA (20 mg/kg) was administered via bolus tail vein injection. Mice were euthanized 21 days after the last (*i.e.*, 4^th^) rhGAA administration and tissue glycogen levels were measured as described in ‘Materials and Methods’. Dotted lines show glycogen levels in the respective tissues of wild-type C57BL/6 mice. The data presented are an average of two independent studies with each bar representing the mean±SEM of the activity measured from 12 mice per group. Statistically significant reductions were seen in glycogen levels compared to baseline (*p<0.05, t-test) and compared to rhGAA administration alone (#p<0.05, t-test). In addition, the effect of AT2220 co-administration was also found to be significant for a linear trend (p<0.05; except in triceps), indicating a dose-dependent effect.

**Figure 6:**
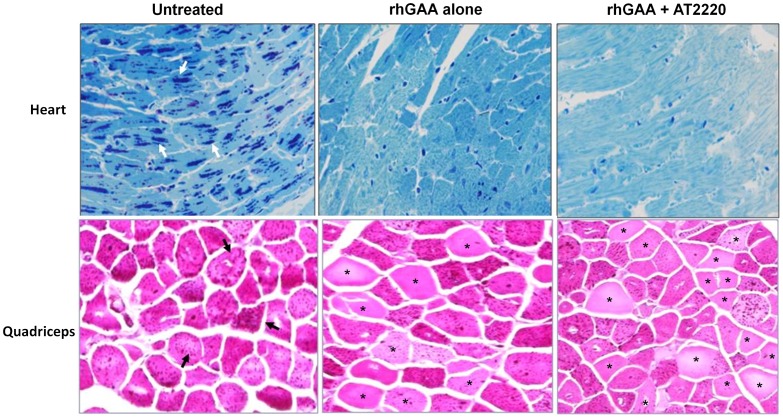
Co-administration of AT2220 promotes cell type-specific reduction of glycogen in GAA KO mice. Twelve-week old male *GAA* KO mice were administered vehicle (water) or AT2220 (30 mg/kg) via oral gavage once every other week for 8 weeks. Thirty minutes after each AT2220 oral administration, vehicle (saline) or rhGAA (20 mg/kg) was administered via bolus tail vein injection. Mice were euthanized 21 days after the last (*i.e.,* 4^th^) rhGAA administration and glycogen levels in heart and quadriceps were measured immunohistochemically as described in ‘Materials and Methods’. A strong glycogen signal, represented as dark blue or pink spots (denoted with arrows) in heart and quadriceps, respectively, was observed. (*) indicates glycogen reduction in individual skeletal muscle fibers of quadriceps. The data shown are representative photomicrographs from 6 mice/group (magnification: 20X).

### Measurement of Plasma GAA Levels

For plasma GAA activity measurements, samples were diluted 400-fold with Lysis Buffer (1% Triton X-100, 150 mM NaCl, 25 mM Bis-Tris, pH 6.5) prior to assay. Plasma (20 µL) was added to 50 µL Assay Buffer (3 mM 4-MUG in 50 mM potassium acetate, pH 4.0) and incubated for 1 hour at 37°C. Reactions were stopped by addition of 70 µL 0.4 M glycine, pH 10.8. Fluorescence at 460 nm was read on a Victor^3^ plate reader after excitation at 355 nm. Raw fluorescence counts were background subtracted (defined by Assay Buffer only). A 4-MU standard curve ranging from 7 nM to 15 µM was run each day for conversion of fluorescence counts to absolute GAA activity, expressed as nanomoles of released 4-MU per milliliter of plasma per hour (nmol/mL/hr). For Western blotting, diluted plasma (∼10 µg total protein) was subjected to SDS-PAGE on 12% polyacrylamide gels (Bio-Rad, Hercules, CA), transferred to PVDF membranes (Bio-Rad), and immunoblotted with the rabbit anti-human GAA primary antibody FL059 (1∶1000 dilution). Protein bands were detected using peroxidase-conjugated goat anti-rabbit secondary antibody in combination with enhanced chemiluminescence (Pierce, Rockford, IL). Blots were scanned on an Image Station 4000R (Kodak, Rochester, NY).

### Measurement of Tissue GAA Levels

Tissue lysates were prepared by homogenization of ∼50 mg tissue for 3 to 5 seconds on ice with a micro-homogenizer (Pro Scientific, Thorofare, NJ) in 200 µL Lysis Buffer. Lysates (20 µL) were added to 50 µL Assay Buffer as described above. A Micro BCA Protein Assay (Pierce) was used to determine total protein concentration in tissue lysates according to the manufacturer’s instructions. A 4-MU standard curve was run each day, and GAA activity was measured as described above and is expressed as nanomoles of released 4-MU per milligram of total protein per hour (nmol/mg protein/hr).

### Measurement of Tissue Glycogen Levels

Tissue glycogen levels were measured as described previously [30] with slight modifications. Briefly, tissue lysates from *GAA* KO and wild-type mice were prepared by homogenization of ∼50 mg tissue for 3 to 5 seconds on ice with a micro-homogenizer in 200 µL Lysis Buffer. Lysates were heat denatured at 99°C for 10 minutes and centrifuged for 10 minutes at 4°C. Supernatants from *GAA* KO and wild-type mice were diluted 1∶10 and 1∶2, respectively, with Lysis Buffer. Diluted lysates (40 µL) were incubated in duplicate with or without 10 µL of 800 U/mL amyloglucosidase for 1 hour at 50°C. The reactions were stopped by heat inactivation at 99°C for 10 minutes, followed by cooling at 4°C for 1 hour. Finally, 200 µL Glucose Assay Reagent was added and absorbance at 340 nm was measured on a Spectramax M2e (Molecular Devices; Sunnyvale, CA). A glycogen standard curve ranging from 5 to 400 µg/mL was run each day for conversion of absorbance data to absolute glycogen levels. Protein levels were measured in lysates (before denaturing) using the Micro BCA Protein Assay Kit according to the manufacturer’s instructions. Data were expressed as micrograms of glycogen cleaved/milligram of protein (µg/mg protein).

### Histochemical Detection of Glycogen

Glycogen levels in heart and quadriceps were determined histochemically using two different types of tissue processing and PAS staining methods. For heart, tissues were fixed, processed, and embedded in Epon-Araldite according to the protocol described previously [31]. Staining was performed using a Periodic Acid-Schiff (PAS) Kit (Sigma) according to the manufacturer’s instructions. Briefly, 1 µm sections were cut and mounted on slides. Sections were hydrated in deionized water for 5 minutes before immersion in 1% periodic acid for 5 minutes. After a brief rinse in deionized water, slides were left in Schiff’s reagent for 15 minutes, rinsed under running tap water for 5 minutes, and air dried. Counterstaining was performed with a 1∶10 dilution of Richardson stain at 45°C for 30 seconds. Sections were washed in deionized water, air dried, and mounted in Acrytol (Surgipath Medical Industries, Richmond IL).

Quadriceps were fixed in 3% glutaraldehyde for 48 hours at 4°C and post-fixed in 1% periodic acid/neutral-buffered formalin (48 hours, 4°C) before processing and paraffin embedding. Tissues were sectioned at 5 µm on an HM 325 microtome (MICROM International, GmbH, Walldorf, Germany). Sections were then dewaxed in xylene and treated with dimedone to reduce background staining during the subsequent PAS reaction. Finally, sections were counterstained in Mayer’s hematoxylin, cleared in xylene, and mounted in Acrytol (Surgipath Medical Industries).

### Data Analysis

Determinations of statistical significance were conducted using Excel 2003 (Microsoft, Redmond, WA) or GraphPad Prism, version 5 (San Diego, CA) as defined in the figure and table legends. Linear trends for dose-dependence were calculated using a one-way ANOVA in GraphPad Prism. The half-life of rhGAA in plasma was calculated using a non-linear one-phase exponential decay curve fitting function in GraphPad Prism.

## Results

### AT2220 Stabilizes rhGAA, Preventing Denaturation and Loss of Activity

The effect of AT2220 binding on the stability of rhGAA was assessed using a fluorescence-based denaturation assay described previously [28]. At 37°C, rhGAA was significantly less stable at neutral pH than at acidic pH, with increases in SYPRO Orange fluorescence occurring over 24 hours (**Fig. 1A**). Importantly, co-incubation with 50 µM AT2220 at neutral pH significantly stabilized rhGAA and prevented denaturation for up to 24 hours, similar to observations made with the apo-enzyme at acidic pH (**Fig. 1A**). Furthermore, incubation of rhGAA at neutral pH/37°C resulted in a time-dependent loss of GAA *activity*, with a half-life of 3 to 4 hours (**Fig. 1B**). In contrast, incubation in acidic buffer, or in neutral pH buffer containing 50 µM AT2220, prevented loss of rhGAA activity for up to 24 hours (**Fig. 1B**). Similarly, incubation of rhGAA in human whole blood resulted in a time-dependent loss of activity; co-incubation with 50 µM AT2220 stabilized the enzyme and prevented loss of activity for up to 24 hours (**Fig. 1C**). Taken together, these results demonstrate that AT2220 stabilizes rhGAA, preventing pH-, temperature-, and time-dependent denaturation and inactivation.

### AT2220 Co-administration Increases the Circulating Half-life of rhGAA in Rats

The effect of AT2220 co-administration on the stability of rhGAA *in vivo* was assessed in 8-week old Sprague Dawley rats. A single oral administration of either 3 or 30 mg/kg AT2220 (which yield C_max_ values of approximately 4 and 40 µM, and are comparable to the C_max_ values seen in humans following oral administration of 50 and 600 mg, respectively [29]), was given 30 minutes prior to bolus intravenous administration of rhGAA to maximize the physical interaction of the two in the circulation (*i.e.,* at AT2220 T_max_). In the absence of AT2220, rhGAA activity declined rapidly in plasma, with a half-life of approximately 1.4 hours (**Fig. 2A** **, upper panel**). In contrast, oral administration of 3 or 30 mg/kg AT2220 prior to rhGAA resulted in a dose-dependent increase in the circulating half-life of rhGAA to 2.1 and 3.0 hours, respectively (**Fig. 2A** **, upper panel**). Western blotting of select time points indicated that AT2220 oral pre-administration leads to greater rhGAA protein levels in plasma compared to rhGAA administration alone. Co-administration of AT2220 increased the quantity of the 110 kDa GAA protein band, with the effect being more pronounced at the 8- and 24-hour time points. Importantly, rhGAA protein was undetectable 24 hours after administration of rhGAA alone, whereas AT2220 pre-administration led to a substantial increase in circulating rhGAA protein at this time point (**Fig. 2A** **, lower panel**).

To more closely mimic the clinical setting, the effect of oral administration of AT2220 prior to rhGAA was also investigated in rats using a 60-minute *intravenous infusion* of rhGAA. In the absence of AT2220, rhGAA showed a half-life of approximately 1.1 hours **(** **Fig. 2B** **, upper panel)**. Oral administration of 3 or 30 mg/kg AT1001 30 minutes prior to the rhGAA infusion resulted in dose-dependent and significant increases of 1.4- and 2.0-fold, respectively, in the circulating half-life of rhGAA **(** **Fig. 2B** **, upper panel)**. A similar effect was seen on rhGAA protein levels following oral pre-administration of AT2220, with the greatest effects seen during the elimination phase (**Fig. 2B** **, lower panel**).

The effect of oral administration of AT2220 on rhGAA was also investigated in *GAA* KO mice. Twelve-week old male *GAA* KO mice were pre-administered AT2220 (30 mg/kg) every other week for 8 weeks. Thirty minutes after each AT2220 oral administration, rhGAA (20 mg/kg) was administered via bolus tail vein injection. Plasma samples were collected 0, 1, and 4 hours after the last (*i.e.,* 4^th^) rhGAA administration for measurement of GAA activity. Similar to the observations in rats, AT2220 co-administration to *GAA* KO mice led to increases in plasma rhGAA activity that were 2.5- and 2.0-fold greater than those seen following administration of rhGAA alone at the 1 and 4 hour time points, respectively (**Fig. 3** **, upper panel**). Concomitantly, an increase in rhGAA protein levels was also seen in plasma (**Fig. 3** **, lower panel)**. Collectively, these data indicate that AT2220 co-administration increases the circulating levels of rhGAA in rats and in *GAA* KO mice.

### Co-administration of AT2220 Increases the Tissue Uptake of rhGAA in GAA KO Mice

Preliminary studies demonstrated that a single bolus tail vein injection of rhGAA results in a dose-dependent increase in GAA activity in disease-relevant tissues of *GAA* KO mice 7 days post-administration, with 40 mg/kg rhGAA showing the greatest effects (**Fig. S1**). Subsequently, repeat-administration studies were conducted in *GAA* KO mice in the absence and presence of 30 mg/kg AT2220, a dose that yields a C_max_ of ∼40 µM, similar to that seen in humans following oral administration of 600 mg AT2220 [29]. Twelve-week old male *GAA* KO mice were administered either vehicle (water) or AT2220 via oral gavage once every other week for 8 weeks. Thirty minutes later, vehicle (saline) or rhGAA (20 mg/kg; the recommended clinical dose) was administered via bolus tail vein injection. Mice were euthanized 7 days after the final (4^th^) administration of rhGAA and tissue GAA levels were measured. Oral pre-administration of AT2220 resulted in increased GAA activity that was 2.5-, 2.2-, 1.5-, 1.7-, 1.7-, and 2.0-fold greater in heart, diaphragm, gastrocnemius, quadriceps, triceps, and tongue, respectively, compared to administration of rhGAA alone (**Fig. 4**).

### Co-administration of AT2220 Leads to Greater Tissue Glycogen Reduction in GAA KO Mice

Next, we determined the effect of AT2220 co-administration on rhGAA-mediated glycogen reduction. Twelve-week old male *GAA* KO mice were pre-administered either vehicle (water) or AT2220 (10 or 30 mg/kg) via oral gavage once every other week for 8 weeks. Thirty minutes after each oral administration of AT2220, vehicle (saline) or rhGAA (20 mg/kg) was administered via bolus tail vein injection. Mice were euthanized 21 days after the final (4^th^) administration of rhGAA and glycogen levels were measured. Co-administration resulted in significantly greater tissue glycogen reduction compared to rhGAA administration alone that was generally dose-dependent (**Fig. 5** **)**. Importantly, co-administration with AT2220 resulted in maximally 1.6-, 1.3-, 2.6-, 2.0-, 2.4-, and 1.4-fold greater glycogen reduction in heart, diaphragm, quadriceps, gastrocnemius, triceps, and tongue, respectively, compared to rhGAA administration alone (**Table 2**). Notably, 20 mg/kg rhGAA co-administered with 30 mg/kg AT2220 resulted in glycogen reductions that were comparable to or greater than those reported previously following administration of 40 mg/kg rhGAA alone [32]. Furthermore, immunohistochemical measurements confirmed that co-administration of 30 mg/kg AT2220 with 20 mg/kg rhGAA results in greater substrate reduction as detected by reduced glycogen in individual smooth muscle and skeletal muscle fibers of the heart and quadriceps, respectively (**Fig. 6**).

**Table 2:** Effect of AT2220 co-administration on rhGAA-mediated glycogen reduction (% change from Baseline) in *GAA* KO mice.

Tissues	*−AT2220*	*+AT2220* *(10 mg/kg)*	*+AT2220 (30 mg/kg)*
**Heart**	−54±5*	−68±5*	−85±2*^#^
**Diaphragm**	−48±6*	−49±5*	−60±6*^#^
**Quadriceps**	−20±4*	−45±3*^#^	−52±4*^#^
**Gastrocnemius**	−21±7	−41±4*	−45±5*^#^
**Triceps**	−10±4	−33±3*^#^	−24±3*^#^
**Tongue**	−44±3*	−54±6*^#^	−62±4*^#^

Twelve-week old male *GAA* KO mice were administered vehicle (water) or AT2220 via oral gavage once every other week for 8 weeks. Thirty minutes after each AT2220 oral administration, rhGAA (20 mg/kg) was administered via bolus tail vein injection. Mice were euthanized 21 days after the last (*i.e.,* 4^th^) rhGAA administration, and tissue glycogen levels were measured as described in ‘Materials and Methods’. Baseline glycogen levels in untreated *GAA* KO mice were 417±32, 340±24, 405±27, 353±21, 446±18, and 389±10 µg/mg protein in heart, diaphragm, quadriceps, gastrocnemius, triceps, and tongue, respectively, and in wild-type C57BL/6 mice were 23±2, 50±6, 40±5, 45±2, 35±7, and 32±2 µg/mg protein, respectively (mean±SEM of 7 mice). The data shown represent the percent glycogen change from baseline in each tissue as normalized between wild-type (0%) and *GAA* KO (100%) levels. Each value represents the mean±SEM of 12 mice. Statistically significant reductions were seen in glycogen levels compared to baseline (*p<0.05, t-test) and compared to rhGAA administration alone (#p<0.05, t-test).

## Discussion

Regular infusion of rhGAA is currently the primary treatment for Pompe disease. However, rhGAA has some limitations including a short circulating half-life [33], inefficient uptake into key tissues [14]–[15], [34], and generation of immune responses that can affect tolerability and efficacy [17]. In addition to the above limitations, our prior [22] and current studies demonstrate that at body temperature, rhGAA is significantly less stable at neutral pH than at acidic pH, which can lead to rapid loss of activity. This instability at neutral pH is noteworthy, but not surprising given that the endogenous enzyme is a resident lysosomal hydrolase with a reported lysosomal half-life of ∼10 days [35]. AT2220 is a PC that has been shown to selectively bind endogenous GAA, increasing its physical stability, lysosomal trafficking, and activity in cultured cells [21]–[22]. Our current studies indicate that the binding of AT2220 to *exogenous* rhGAA significantly increases the physical stability and prevents denaturation of the enzyme at 37 °C in neutral pH buffer *in vitro* and in human blood *ex vivo*. The binding and stabilization of rhGAA by a small molecule PC may explain the increase in rhGAA cellular levels and tissue uptake that were reported in a previous study that utilized the AT2220 derivative *N*-butyl-DNJ in combination with rhGAA [24]. Recently, PCs have also been shown to increase the stability and to improve the cellular and tissue uptake of two other exogenous recombinant enzymes that are used to treat LSDs, namely recombinant human acid β-glucosidase [27] and α-galactosidase A [24]–[26]. In the case of α-galactosidase A, greater substrate reduction was also realized when used in combination with a PC [25]–[26].

Similar to the stabilizing effects seen in blood *ex vivo*, AT2220 co-administration to mice and rats also prolonged the half-life of rhGAA in the circulation. Importantly, these effects were seen at doses (3 and 30 mg/kg) that result in plasma C_max_ concentrations of approximately 4 and 40 µM in rodents, which are comparable to those achieved in humans following oral administration of 50 and 600 mg AT2220, respectively [29]. Furthermore, AT2220 co-administration also resulted in up to 2.5-fold higher levels of GAA activity in disease-relevant tissues of *GAA* KO mice compared to administration of rhGAA alone. We hypothesize that the binding of AT2220 to rhGAA may sufficiently increase the physical stability of the exogenous enzyme to minimize or prevent thermally- and neutral pH-mediated denaturation, as well as proteolysis, in the blood. A longer circulating half-life of the stabilized, properly folded, functional enzyme may increase the likelihood for recognition by cation-independent mannose 6-phosphate receptors (CI-MPRs) and subsequent uptake into disease-relevant cells and tissues leading to improved substrate reduction. In addition, AT2220-mediated stabilization of rhGAA in lysosomes, vesicles, and other non-lysosomal compartments that are involved in the endocytic pathway could lead to less or slower intracellular degradation of rhGAA, and hence higher cellular/tissue levels. Importantly, co-localization studies with fluorescent rhGAA and LAMP2 have shown that PCs can improve the delivery of both endogenous and exogenous GAA to lysosomes, resulting in higher cellular levels of the fully-processed, mature forms of GAA [22], [24]. These mature, lysosomal forms with molecular weights of 70 and 76 kDa more effectively catalyze glycogen turnover compared to the 110 kDa precursor form [36]–[37].

Notably, co-administration of AT2220 and rhGAA lead to greater glycogen reduction in tissues of *GAA* KO mice. Whereas four injections of 20 mg/kg rhGAA showed little effect on the glycogen levels of some skeletal muscles such as gastrocnemius and triceps, co-administration with AT2220 resulted in significant reductions. Furthermore, co-administration of 20 mg/kg rhGAA with AT2220 resulted in glycogen reductions that were similar to those reported previously with 40 mg/kg rhGAA alone [32], suggesting that AT2220 can improve the potency of rhGAA.

While glycogen reduction was significantly greater following co-administration of AT2220 and rhGAA, complete correction has yet to be achieved (*i.e.,* tissue glycogen levels do not reach the levels seen in wild-type mice). This may be due to a number of factors. First, wild-type tissue GAA levels are not realized even upon co-administration of rhGAA and AT2220 at the doses tested. In fact, total GAA tissue levels achieved with co-administration of AT2220 were maximally 30% to 60% of wild-type levels for a limited amount of time. Second, it has been shown that rhGAA is often mistrafficked in Pompe cells due to abnormal recycling of the CI-MPR [15]–[16], which is essential for rhGAA uptake and delivery to late endosomal/lysosomal compartments. Hence, while more rhGAA is taken up into cells and tissues, and higher lysosomal levels are achieved, it is possible that some of the exogenous enzyme is delivered to inappropriate cellular compartments (*i.e.,* not lysosomes), and hence is not accessible to some of the glycogen pools [16]. Third, current commercial preparations of rhGAA have poor affinity for the CI-MPR due to low mannose 6-phosphate (Man6-P) content [32], [38]. This, combined with a reduced abundance of the CI-MPR in skeletal muscles of Pompe mice [39], may limit delivery of rhGAA to lysosomes. Currently, we do not know if AT2220 can increase the affinity of rhGAA for the CI-MPR. However, a new form of rhGAA that is conjugated to synthetic oligosaccharides that carry high levels of Man6-P (oxime-neo-rhGAA) showed increased affinity for the CI-MPR, and approximately 5-fold greater efficacy for reducing glycogen compared to the unmodified form [38]. Evaluation of this chemically-modified form of rhGAA, or other forms with modified targeted motifs (NCT01435772; NCT01230801), in combination with AT2220 is warranted to determine if increased physical stability of high-affinity rhGAA may further improve tissue uptake and glycogen reduction in *GAA* KO mice. Lastly, rhGAA is highly immunogenic, which may impact its activity *in vivo*.

To this end, repeat administrations of rhGAA to *GAA* KO mice leads to the development of a severe immune response due to the development of anti-GAA antibodies. Due to the severity of the immune response in these mice, the duration of the studies are limited to four rhGAA administrations or less (due to mortalities that arise beginning with the third rhGAA administration), again potentially affecting the maximum long-term efficacy that can be realized in a preclinical setting. An established line of immune-tolerant mice [40] or a recently developed transgenic mouse model that expresses low levels of a PC-responsive mutant form of human GAA (P545L) on a *GAA* KO background [41] can be used for future long-term rhGAA efficacy studies in combination with AT2220.

Similar to the observations in mice, the cross-reactive immunologic material (CRIM) status of Pompe patients can substantially influence the efficacy and/or tolerability of ERT in the clinic [17]. Pompe patients who do not produce native enzyme (referred to as CRIM-negative) are more prone to develop a sustained immune response with high anti-GAA antibody titers (some of which can be neutralizing), compared to CRIM-positive patients [18]. Treatment of patients with immunomodulatory agents such as methotrexate has led to improved ERT-treatment outcomes in CRIM-negative Pompe patients [42] and in mice [43]; similarly, genetically- or chemically-induced immune tolerance has been shown to significantly reduce IgG levels in *GAA* KO mice [44]–[46]. Furthermore, misfolded, denatured, and/or aggregated therapeutic proteins are known to be more immunogenic than correctly folded, stable protein therapeutics [47]. Thus, it is possible that the ERT-mediated immunogenicity observed in many Pompe patients results from destabilization or denaturation of rhGAA in the blood (and possibly in the infusion solution). By stabilizing rhGAA in its correctly folded, monomeric, native conformation, we hypothesize that AT2220 co-administration may attenuate rhGAA-mediated immunogenicity. Future repeat-administration studies focused on measuring IgG levels with rhGAA in combination with AT2220 in *GAA* KO mice will be needed to further investigate these possibilities.

Recent studies indicate that there is also a neuropathological component to Pompe disease that is characterized by widespread CNS pathology [48]–[50]. However, motoneurons seem particularly susceptible to glycogen accumulation in both patients and *GAA* KO mice, and reduced motor output has been described [48]–[50]. Though AT2220-mediated increases in ERT penetration into the central nervous system are unlikely, it is possible that co-administration could lead to greater rhGAA uptake into neurons of the peripheral nervous system (*e.g.,* phrenic nerves and sensory ganglia) [48], potentially leading to greater glycogen reduction and improved neuronal function. Future studies will be necessary to evaluate this potential, particularly on phrenic nerve pathology and overall respiratory function.

Collectively, our data indicate that AT2220 increases the physical stability of rhGAA, and leads to higher cellular and tissue activity as evidenced by greater substrate turnover *in situ*. As such, co-administration may provide an improved therapeutic strategy for the treatment of Pompe disease. Based on these encouraging findings, a Phase 2 clinical study has been initiated to investigate the combination of AT2220 with rhGAA in Pompe patients.

## Supporting Information

Figure S1 **Dose-dependent increases in tissue GAA activity following administration of rhGAA to GAA KO mice.** Twelve-week old male *GAA* KO mice were administered 10, 20, or 40 mg/kg rhGAA via bolus tail vein injection. Mice were euthanized 7 days later, and GAA activity was measured in disease-relevant tissues (see “Materials and Methods” in the original article). Administration of rhGAA led to dose-dependent and significant increases (*p<0.05 compared to baseline, via t-test) in tissue GAA levels, with the greatest activity seen with the 40 mg/kg dose. Each bar represents the mean±SEM for 4–7 mice per group.
(TIF)

## References

[1] Hirschhorn R, Reuser AJJ, Scriver C, Beaudet A, Sly W, Valle D (2001). Glycogen storage disease type ii: Acid alpha-glucosidase (acid maltase) deficiency..

[2] van der Ploeg AT, Reuser AJJ (2008). Pompe's disease.. Lancet.

[3] van den Hout HM, Hop W, van Diggelen OP, Smeitink JA, Smit GP (2003). The natural course of infantile pompe's disease: 20 original cases compared with 133 cases from the literature.. Pediatrics.

[4] Kishnani PS, Howell RR (2004). Pompe disease in infants and children.. J Pediatr.

[5] Kishnani PS, Nicolino M, Voit T, Rogers RC, Tsai AC (2006). Chinese hamster ovary cell-derived recombinant human acid alpha-glucosidase in infantile-onset pompe disease.. J Pediatr.

[6] Van den Hout H, Reuser AJ, Vulto AG, Loonen MC, Cromme-Dijkhuis A (2000). Recombinant human alpha-glucosidase from rabbit milk in pompe patients.. Lancet.

[7] Van den Hout JM, Kamphoven JH, Winkel LP, Arts WF, De Klerk JB (2004). Long-term intravenous treatment of pompe disease with recombinant human alpha-glucosidase from milk.. Pediatrics.

[8] Nicolino M, Byrne B, Wraith JE, Leslie N, Mandel H (2009). Clinical outcomes after long-term treatment with alglucosidase alfa in infants and children with advanced pompe disease.. Genet Med.

[9] Chen L-R, Chen C-A, Chiu S-N, Chien Y-H, Lee N-C (2009). Reversal of cardiac dysfunction after enzyme replacement in patients with infantile-onset pompe disease.. J Pediatr.

[10] Strothotte S, Strigl-Pill N, Grunert B, Kornblum C, Eger K (2010). Enzyme replacement therapy with alglucosidase alfa in 44 patients with late-onset glycogen storage disease type 2: 12-month results of an observational clinical trial.. J Neurol.

[11] van der Ploeg AT, Clemens PR, Corzo D, Escolar DM, Florence J (2010). A randomized study of alglucosidase alfa in late-onset pompe's disease.. N Engl J Med.

[12] Thurberg BL, Lynch Maloney C, Vaccaro C, Afonso K, Tsai AC-H (2006). Characterization of pre- and post-treatment pathology after enzyme replacement therapy for pompe disease.. Lab Invest.

[13] Schoser B, Hill V, Raben N (2008). Therapeutic approaches in glycogen storage disease type ii/pompe disease.. Neurotherapeutics.

[14] Raben N, Danon M, Gilbert AL, Dwivedi S, Collins B (2003). Enzyme replacement therapy in the mouse model of pompe disease.. Mol Genet Metab.

[15] Fukuda T, Roberts A, Ahearn M, Zaal K, Ralston E (2006). Autophagy and lysosomes in pompe disease.. Autophagy.

[16] Cardone M, Porto C, Tarallo A, Vicinanza M, Rossi B (2008). Abnormal mannose-6-phosphate receptor trafficking impairs recombinant alpha-glucosidase uptake in pompe disease fibroblasts.. Pathogenetics.

[17] Kishnani PS, Goldenberg PC, DeArmey SL, Heller J, Benjamin D (2010). Cross-reactive immunologic material status affects treatment outcomes in pompe disease infants.. Mol Genet Metab.

[18] de Vries JM, van der Beek NAME, Kroos MA, Özkan L, van Doorn PA (2010). High antibody titer in an adult with Pompe disease affects treatment with alglucosidase alfa.. Mol Genet Metab.

[19] Fan J-Q (2008). A counterintuitive approach to treat enzyme deficiencies: Use of enzyme inhibitors for restoring mutant enzyme activity.. Biol Chem.

[20] Okumiya T, Kroos MA, Vliet LV, Takeuchi H, Van der Ploeg AT (2007). Chemical chaperones improve transport and enhance stability of mutant alpha-glucosidases in glycogen storage disease type ii.. Mol Genet Metab.

[21] Parenti G, Zuppaldi A, Gabriela Pittis M, Rosaria Tuzzi M, Annunziata I (2007). Pharmacological enhancement of mutated alpha-glucosidase activity in fibroblasts from patients with pompe disease.. Mol Ther.

[22] Flanagan JJ, Rossi B, Tang K, Wu X, Mascioli K (2009). The pharmacological chaperone 1-deoxynojirimycin increases the activity and lysosomal trafficking of multiple mutant forms of acid alpha-glucosidase.. Hum Mutat.

[23] Valenzano KJ, Khanna R, Powe AC, Boyd R, Lee G (2011). Identification and characterization of pharmacological chaperones to correct enzyme deficiencies in lysosomal storage disorders.. Assay Drug Dev Technol.

[24] Porto C, Cardone M, Fontana F, Rossi B, Tuzzi MR (2009). The pharmacological chaperone n-butyldeoxynojirimycin enhances enzyme replacement therapy in pompe disease fibroblasts.. Mol Ther.

[25] Porto C, Pisani A, Rosa M, Acampora E, Avolio V (2012). Synergy between the pharmacological chaperone 1-deoxygalactonojirimycin and the human recombinant alpha-galactosidase a in cultured fibroblasts from patients with fabry disease.. J Inherit Metab Dis.

[26] Benjamin ER, Khanna R, Schilling A, Flanagan JJ, Pellegrino LJ (2012). Co-administration with the pharmacological chaperone at1001 increases recombinant human alpha-galactosidase a tissue uptake and improves substrate reduction in fabry mice.. Mol Ther.

[27] Shen J-S, Edwards NJ, Hong YB, Murray GJ (2008). Isofagomine increases lysosomal delivery of exogenous glucocerebrosidase.. Biochem Biophys Res Commun.

[28] Niesen FH, Berglund H, Vedadi M (2007). The use of differential scanning fluorimetry to detect ligand interactions that promote protein stability.. Nat Protocols.

[29] Adera M, Boudes P, Bragat A, Sitaraman S, Lazauskas R (2011). Pharmacokinetics and muscle distribution of at2220, a pharmacological chaperone of acid-glucosidase, in healthy volunteers.. Mol Genet Metab.

[30] Amalfitano A, McVie-Wylie AJ, Hu H, Dawson TL, Raben N (1999). Systemic correction of the muscle disorder glycogen storage disease type ii after hepatic targeting of a modified adenovirus vector encoding human acid-alpha-glucosidase.. Proc Natl Acad Sci USA.

[31] Lynch CM, Johnson J, Vaccaro C, Thurberg BL (2005). High-resolution light microscopy (hrlm) and digital analysis of pompe disease pathology.. J Histochem Cytochem.

[32] Zhu Y, Li X, McVie-Wylie A, Jiang C, Thurberg BL (2005). Carbohydrate-remodelled acid alpha-glucosidase with higher affinity for the cation-independent mannose 6-phosphate receptor demonstrates improved delivery to muscles of pompe mice.. Biochem J.

[33] Myozyme package insert (2006). Alglucosidase alfa. Cambridge, MA: Genzyme Corp.. http://www.myozyme.com/~/media/Files/MyozymeUS/Documents/mz_pi.pdf.

[34] Drost MR, Schaart G, Dijk Pv, Capelle CIv, Vusse GJvD (2008). Both type 1 and type 2a muscle fibers can respond to enzyme therapy in pompe disease.. Muscle Nerve.

[35] Reuser AJJ, Kroos MA, Ponne NJ, Wolterman RA, Loonen MCB (1984). Uptake and stability of human and bovine acid [alpha]-glucosidase in cultured fibroblasts and skeletal muscle cells from glycogenosis type ii patients.. Exp Cell Res.

[36] Wisselaar HA, Kroos MA, Hermans MM, van Beeumen J, Reuser AJ (1993). Structural and functional changes of lysosomal acid alpha-glucosidase during intracellular transport and maturation.. J Biol Chem.

[37] Bijvoet AG, van de Kamp EH, Kroos MA, Ding JH, Yang BZ (1998). Generalized glycogen storage and cardiomegaly in a knockout mouse model of pompe disease.. Hum Mol Genet.

[38] Zhu Y, Jiang J-L, Gumlaw NK, Zhang J, Bercury SD (2009). Glycoengineered acid alpha-glucosidase with improved efficacy at correcting the metabolic aberrations and motor function deficits in a mouse model of pompe disease.. Mol Ther.

[39] Koeberl DD, Luo X, Sun B, McVie-Wylie A, Dai J (2011). Enhanced efficacy of enzyme replacement therapy in pompe disease through mannose-6-phosphate receptor expression in skeletal muscle.. Mol Genet Metab.

[40] Raben N, Nagaraju K, Lee A, Lu N, Rivera Y (2003). Induction of tolerance to a recombinant human enzyme, acid alpha-glucosidase, in enzyme deficient knockout mice.. Transgenic Res.

[41] Khanna R, Soksa R, Feng J, Lun Y, Powe AC (2009). G.P.8.05 the pharmacological chaperone at2220 increases mutant acid alpha-glucosidase levels and reduces tissue glycogen in a mouse model of pompe disease.. Neuromuscul Disord.

[42] Mendelsohn NJ, Messinger YH, Rosenberg AS, Kishnani PS (2009). Elimination of antibodies to recombinant enzyme in pompe's disease.. N Engl J Med.

[43] Sun B, Kulis MD, Young SP, Hobeika AC, Li S (2009). Immunomodulatory gene therapy prevents antibody formation and lethal hypersensitivity reactions in murine pompe disease.. Mol Ther.

[44] Joseph A, Munroe K, Housman M, Garman R, Richards S (2008). Immune tolerance induction to enzyme-replacement therapy by co-administration of short-term, low-dose methotrexate in a murine pompe disease model.. Clin Exp Immunol.

[45] Douillard-Guilloux G, Mouly V, Caillaud C, Richard E (2009). Immortalization of murine muscle cells from lysosomal alpha-glucosidase deficient mice: A new tool to study pathophysiology and assess therapeutic strategies for pompe disease.. Biochem Biophys Res Commun.

[46] van Til NP, Stok M, Aerts Kaya FSF, de Waard MC, Farahbakhshian E (2010). Lentiviral gene therapy of murine hematopoietic stem cells ameliorates the pompe disease phenotype.. Blood.

[47] Thai R, Moine G, Desmadril M, Servent D, Tarride J-L (2004). Antigen stability controls antigen presentation.. J Biol Chem.

[48] DeRuisseau LR, Fuller DD, Qiu K, DeRuisseau KC, Donnelly WH (2009). Neural deficits contribute to respiratory insufficiency in pompe disease.. Proceedings of the National Academy of Sciences.

[49] Sidman RL, Taksir T, Fidler J, Zhao M, Dodge JC (2008). Temporal neuropathologic and behavioral phenotype of 6neo/6neo pompe disease mice.. J Neuropathol Exp Neurol.

[50] Lee C-J, Fan X, Guo X, Medin JA (2011). Promoter-specific lentivectors for long-term, cardiac-directed therapy of fabry disease.. J Cardiol.

