# Supplementary figures and images for: The Pharmacological Chaperone AT2220 Increases Recombinant Human Acid α-Glucosidase Uptake and Glycogen Reduction in a Mouse Model of Pompe Disease
Source: PLoS One. 2012 Jul 18;7(7):e40776. doi: 10.1371/journal.pone.0040776 (PMC3399870; doi:10.1371/journal.pone.0040776)

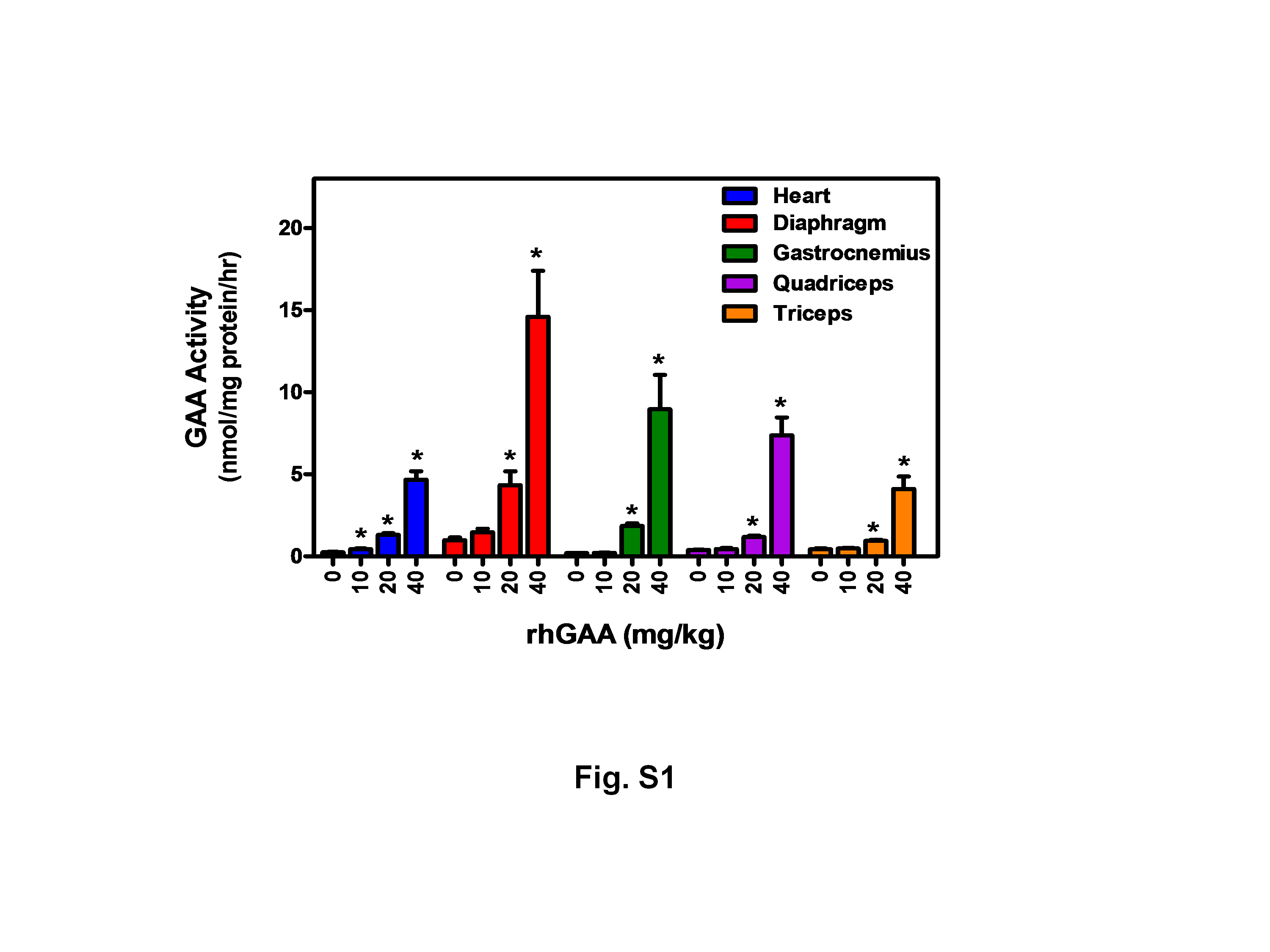

Supplement: Figure S1 — Dose-dependent increases in tissue GAA activity following administration of rhGAA to GAA KO mice. Twelve-week old male GAA KO mice were administered 10, 20, or 40 mg/kg rhGAA via bolus tail vein injection. Mice were euthanized 7 days later, and GAA activity was measured in disease-relevant tissues (see “Materials and Methods” in the original article). Administration of rhGAA led to dose-dependent and significant increases (*p<0.05 compared to baseline, via t-test) in tissue GAA levels, with the greatest activity seen with the 40 mg/kg dose. Each bar represents the mean±SEM for 4–7 mice per group. (TIF) [file pone.0040776.s001.tif]
